# Native aggregation as a cause of origin of temporary cellular structures needed for all forms of cellular activity, signaling and transformations

**DOI:** 10.1186/1742-4682-7-19

**Published:** 2010-06-09

**Authors:** Vladimir V Matveev

**Affiliations:** 1 Laboratory of Cell Physiology, Institute of Cytology, Russian Academy of Sciences, Tikhoretsky Ave 4, St. Petersburg 194064, Russia

## Abstract

According to the hypothesis explored in this paper, native aggregation is genetically controlled (programmed) reversible aggregation that occurs when interacting proteins form new temporary structures through highly specific interactions. It is assumed that Anfinsen's dogma may be extended to protein aggregation: composition and amino acid sequence determine not only the secondary and tertiary structure of single protein, but also the structure of protein aggregates (associates). Cell function is considered as a transition between two states (two states model), the resting state and state of activity (this applies to the cell as a whole and to its individual structures). In the resting state, the key proteins are found in the following inactive forms: natively unfolded and globular. When the cell is activated, secondary structures appear in natively unfolded proteins (including unfolded regions in other proteins), and globular proteins begin to melt and their secondary structures become available for interaction with the secondary structures of other proteins. These temporary secondary structures provide a means for highly specific interactions between proteins. As a result, native aggregation creates temporary structures necessary for cell activity.

"One of the principal objects of theoretical research in any department of knowledge is to find the point of view from which the subject appears in its greatest simplicity."

Josiah Willard Gibbs (1839-1903)

## Introduction

To date, numerous mechanisms, signal pathways, and different factors have been found in the cell. Researchers are naturally eager to find commonalities in the mechanisms of cellular regulation. I would like to propose a substantial approach to problems of cell physiology - the structural ground that produces signals and underlies the diversity of cellular mechanisms.

The methodological basis for the proposed hypothesis results from studies by the scientific schools of Dmitrii Nasonov [1] and Gilbert Ling [2-6], which have gained new appreciation over the last 20-30 years owing to advances in protein physics [7] in the study of properties of globular proteins, their unfolding and folding, as well as the discovery of novel states of the protein molecule: the natively unfolded and the molten globule. The key statement for the rationale of the present paper is that the specificity of interactions of polypeptide chains with each other (at the intra- and inter-molecular levels) can be provided only by their secondary structures, primarily α-helices and β-sheets.

Nasonov's school discovered and studied a fundamental phenomenon -- the nonspecific reaction of the cell to external actions [1], while works by Ling [5] and his followers allow the mechanisms of this phenomenon to be understood.

The above-mentioned cell reaction has been called nonspecific because diverse physical and chemical factors produce the same complex of structural changes in the cell: an increase in the turbidity and macroscopic viscosity of the cytoplasm and in the adsorption of hydrophobic substances by cytoplasmic proteins. It is of primary importance that the same changes also occur in the cell during its transition into the active state: muscle contraction, action potential, enhancement of secretory activity (for details, see [8]). Hence, from the point of view of structural changes, there is no fundamental difference between the result of action on the cell of hydrostatic pressure and, for instance, muscle contraction. In both cases, proteins are aggregated.

Nasonov called the cause of these changes the stages of cell protein denaturation, as the changes of properties of isolated proteins during denaturation are very similar to the changes in the cytoplasm during the nonspecific reaction. As a result, the denaturational theory of cell excitation and damage was created [1]. The structural changes of protein denaturation were unclear in Nasonov's time. Nowadays, it is assumed that the denaturation is the destruction of the tertiary and secondary structure of a protein. Below I give two definitions, for the denaturation of natively folded (globular) proteins and for natively unfolded proteins.

A key notion in physiology is the *resting state *of the cell. This is implicit in the concept of the threshold character of the action of stimuli on the cell, which has played a historical role in the development of physiological science. It is the threshold that is the boundary between two states -- rest and activity. But in effect, all our knowledge about cells concerns active cells, not cells in the resting state. It is in the active cell that variable changes occur that can be recorded. Nothing happens in the resting cell, so there is nothing to be recorded in it. Nevertheless, it is obvious that the resting state is the initial cell state, the starting point for all changes occurring in the cell.

What characterizes the structural aspect of the cell in the state of rest? It is only in Ling's work [5] that I have found a clear answer to this question. The answer can be interpreted as follows: if all resting cell proteins were arranged in one line, it would turn out that most of the peptide bonds in this superpolypeptide would be accessible to solvent (water), while only a few would be included in secondary structures. When the cell is activated, the ratio between the unfolded and folded areas is changed sharply to the opposite: the proportion of peptide bonds accessible to solvent decreases markedly, whereas the proportion included in secondary structures rises significantly. These two extreme states of cell proteins, suggested by Ling, provide a basis for further consideration.

If Ling's approach is combined with Nasonov's theory, we obtain several interesting consequences. First of all, it is clear that proteins with maximally unfolded structures form the structural basis of resting cells because they are inactive, i.e., do not interact with other proteins or other macromolecules. The situation changes when an action on the cell exceeds the threshold: completely or partially unfolded key proteins begin to fold when new secondary protein structures are formed. Owing to these new secondary structures, the proteins become capable of reacting, i.e., intramolecular aggregation (folding of individual polypeptides into globules) and intermolecular aggregation (interaction of some proteins with others) begin. A distinguishing feature of these aggregational processes is their absolutely specific character, which is ensured by the amino acid composition, shape, and size of the secondary structures. The structures appearing have physiological meaning, so such aggregation is native and the secondary structures causing it are centers of native aggregation. Another source of secondary structures necessary for native aggregation is the molten globule.

The ability of cells to return to the initial state, the state of rest, means that native aggregation is completely reversible, and the structures appearing in the course of native aggregation are temporary and are disassembled as soon as they cease to be necessary. Native aggregation can involve both the whole cell and individual organelles, compartments, and structures, and activation of proteins is of a threshold rather than a spontaneous character.

The meaning of the proposed hypothesis of native aggregation is that the primary cause of any functional changes in cell is the appearance, as a result of native aggregation, of *temporary *structures, continually appearing and disintegrating during the life of the cell. Since native aggregation is initiated by external stimuli or regulatory processes and the structures appearing have a temporary character, these structures can be called signal structures.

Signal structures can have different properties: (i) they can be centers of binding of ions, molecules (solutes), and proteins; (ii) they can have enzymatic activity; (iii) they can form channels and intercellular contacts; (iv) they can serve as matrices organizing the interactions of molecules in synthetic and transport processes; (iv) they can serve as receptors for signal molecules; (v) they can serve as the basis for constructing even more complex supramolecular structures. These structures "flash" in the cell space like signal lights, perform their role, and disappear, to appear in another place and at another time. The meaning of the existence of the structural "flashes" is that during transition into the active state the cell needs new resources, functions, mechanisms, regulators, and signals. As soon as the cell changes to the resting state, the need for these structures disappears, and they are disassembled. Extreme examples of native aggregation are muscle contraction, condensation of chromosomes, the appearance of the division spindle, and interactions of ligands with receptors.

Thus, the present paper will consider the meaning and significance of native aggregation as the universal structural basis of the active cell. The basis of pathological states is the inability of the cell to return to the resting state and errors in the formation of signal structures. The presentation of native aggregation is based on three pillars: (i) reversible protein aggregation is a structural basis of cell activity (Nasonov's School); (ii) the operation of the living cell or its individual structures can be regarded as a repetitive sequence of transitions between two states (active and resting), a key role in which belongs to natively unfolded proteins (Ling's approach); (iii) the specificity of interactions of separate parts of a single polypeptide chain with each other (folding) or the interaction of separate polypeptide chains among themselves (self-assembly, aggregation) can be provided only by protein secondary structures.

The goal of this paper is the enunciation of principles, rather than a review of facts corresponding to these principles.

## Native aggregation in retrospective

The best-studied nonspecific response of cells to external actions might possibly be the response to fixatives. For a long time in the history of science, cells were considered optically empty structures by researchers. The appearance of methods of fixation and staining wrought a revolution in cytology, as these approaches opened to the researchers' sight numerous cell structures whose existence had not even been suspected. After a period of euphoria, doubts were cast: were these structures real or were they the results of fixation, *denaturation *of the cell's native substance?

The danger of serious errors when artifacts of fixation might be considered real structures became a subject of general attention after 1899 (see [9], Ch. 1 for details), when coagulation of homogenous protein solutions was shown to lead to the appearance of structures quite similar to those observed in fixed cell preparations (see [10], Fig. twenty-four). The shape of such artificial structures depended on the chemical nature of the fixative, its concentration, the protein concentration in solution, the temperature, and other conditions. This brought about an obvious crisis in the study of cell morphology.

However, other things were also obvious. In the optically empty part of the cell, visible structures could appear not only during fixation, but also during the transition of the cell to the active state. Comparative observations on fixed preparations and living cells showed that where the structure appeared *in vivo*, it was also observed in a fixed preparation. The obvious resemblance between native structures and the structures obtained as a result of fixation gave grounds for considering that several cell structures are formed not only at fixation, but also during activation of some particular cell fraction, when new structures absent in the resting cell are formed by self-assembly (see [9], Ch. 1 for details).

This discussion has led to the rather important conclusion that despite the dangers of producing artifacts, another thing is beyond doubt: in the process of aggregation, the denatured cell proteins interact with each other not chaotically, but regularly, in accordance with a certain plan (this is what I call native aggregation). The laws of this interaction lead to the formation of temporary structures necessary for the cell to function under new conditions. During fixation and dehydration, this process initially occurs "as it should" (the self-assembly of real cellular structures takes place), but it goes too far when the process of making the preparation is completed, when aggregation becomes irreversible and the structure appearing as a result of aggregation becomes a "corpse". If the interaction of proteins during aggregation had been chaotic, we would still know little about cell structure.

The course of native aggregation seems to be determined by the non-homogeneity of the content of the resting cell; it has structure that is invisible under the light microscope, but reveals itself at the onset of native aggregation. The role of structure guiding native aggregation may be played, for instance, by Porter's "microtrabecular lattice" [11], which can be envisaged «...as that which is in the background of all the visible membranous organelles and all the visible elements of the cytoskeleton; e.g., that which has been invisible up until now and which we wish to "see" microscopically» [12]. Such a lattice might act as the center of "crystallization" or the center of "attachment" of aggregating proteins. However, this is merely an example that I cite for clarity. The centers of crystallization can also comprise the most sensitive proteins that are the first to respond by conformational alterations to changes in the medium and become aggregation-competent. In any event, as a result of native aggregation, the hidden structures become visible under the microscope.

Fulton [13], a convinced Porter devotee, moved even further: she put forward a point of view that "the cytoplasm is so compact that it is only occasionally more open than a crystal". Sufficient data have probably accumulated in the literature to establish that the content of a cell is to be considered a structured system that guides native aggregation into the required course. As one example, one can indicate the data of Balό-Banga et al. [14]: the birefringence of lymphocyte nuclei was enhanced after fixation with ethanol, i.e., correct fixation leads to the appearance of new, more ordered structures. However, especially interesting are the cases when native aggregation, as I call it, takes place in the process of normal cell functioning. Thus, in the same work by Balό-Banga et al. [14], activation of lymphocytes by specific antigens or haptens was shown to lead to a significant enhancement of nuclear birefringence. The same phenomenon was also observed in the case of activation of peripheral blood lymphocytes with allergens in drug-allergic patients [15].

If the factor affecting the cell becomes more intense, its activating effect will be replaced by a damaging one. Thus, the studies of Inners and Bendet [16] on thermal DNA denaturation in bacteriophage T2 and spermatozoa [17] showed that during irreversible denaturation of structures their capacity for birefringence is lost. Such data indicate that under certain conditions, the actions of heat, organic solvents, etc. on cells produce not native aggregation, but destruction, disorganization of intracellular structure; in other words, destruction of structure can follow native aggregation. Unfortunately, there is a marked tendency in the literature towards rough alterations in the structure of the cytoplasm and organelles, because they are easier to study.

Thus, the retrospective considered shows that when adequate methods of study are used, native (programmed) protein aggregation leading to self-assembly of various cell structures is the usual phenomenon of cell life. An example of this is the universal reaction of the living cell [8].

## Universal reaction of the living cell and native aggregation

Why does native aggregation not occur in cells in the resting state but begins only on activation (for instance, muscle contraction, action potential) or damage? To answer this question, let us return to Nasonov's denaturation theory [1]. According to this theory, excitation of the cell takes place only when its proteins are subjected to the initial stages of denaturation.

Mirsky seems to have been the first to pay attention to the similarity between changes in active cytoplasm and the denaturation of isolated proteins [18]. Mirsky came to the conclusion that denaturational protein changes appear when an egg cell is fertilized [19] and during photoreception [20]. This is what he says about it in the latter of the above-cited works: "...There is evidence indicating that light denatures a conjugated protein, visual purple, and that denaturation reverses in the dark." However, his studies in this direction were not systematic.

**Figure 1:**
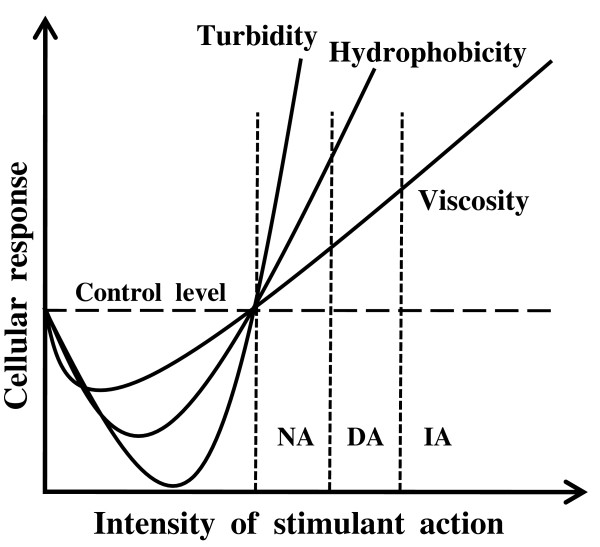
**Response reaction of cell depending on strength of external action (scheme)**. On reaching the threshold, the first phase of the cellular response begins; during this phase the cell becomes more transparent, while hydrophobicity and macroscopic viscosity decrease. Then the second phase of the cellular response begins, during which all parameters measured significantly exceed the control level (in this case, the control level means the cell resting state). NA, native aggregation when necessary cellular functions and signaling pathways are activated; DA, damaging aggregation when signals for apoptosis, cancer transformation or other pathological cellular states may be generated; IA, irreversible aggregation leading to cell death. See [8] for details.

Nasonov and his followers studied the effects of quite different factors (chemical substances, pH, hydrostatic pressure, mechanical action) on cells of different types. As a result, a regularity was revealed: regardless of the character of the action and the type of cell, the response reaction represented a monotypic (nonspecific) complex of *synchronous *changes. These changes were of two-phase character: macroscopic viscosity first decreased, then rose; binding of vital dyes by cell structures (under conditions of diffuse equilibrium) first decreased, then increased; in the first phase of the reaction the cytoplasm became clear, in the second phase it became turbid. Other parameters (see [8] for review) were also studied. The first phase of this reaction is not related to the subject of the present paper, as it is a variation of the resting state. Of interest to us is the second phase, whose structural basis is protein aggregation (Fig. 1). It is this phase that is the phase of activation of cell functions [1].

This second phase was called the phase of excitation and damage by Nasonov's school. Substantial changes in the cell in this phase are remarkably reminiscent of denaturation of isolated proteins; therefore, Nasonov called his theory explaining the cell response reaction the denaturational theory of excitation and damage. According to this theory, the initial stages of denaturational changes, when they still are reversible, underlie cell excitation (activation of secretory function, muscle contraction, action potential, etc.). More profound protein changes lead to disturbances of normal cell functioning, but may still be reversible. Then, with further development of damage, denaturational changes become irreversible and the cell dies.

The peculiarity of the cellular reaction discovered and studied by Nasonov's school was its *nonspecific *character: whatever the action on the cell was, its proteins were aggregated (as in fixation); any cellular activity was also accompanied by protein aggregation (this is especially well seen in the case of muscle contraction). The behavior of isolated proteins during denaturation was the same: any denaturing agent caused their aggregation (except for denaturation under non-physiological conditions, e.g. denaturation by concentrated solutions of urea).

In this universalism of the cellular response, a puzzle was hidden, but in an era concerned with specific interactions, nonspecific phenomena drew no attention. Nevertheless, it is obvious that the nonspecific cellular reaction discovered by Nasonov's school is a fundamental natural phenomenon - like cell division or carcinogenesis. Attention to it is justified because the phenomena of nature, unlike theories, cannot be erroneous.

The nonspecific character of the cellular reaction considered is a superficial impression. Death is also a nonspecific phenomenon, but the processes leading to it are characterized by diversity and can be extremely specific. In exactly the same way, aggregation of proteins can be based on specific interactions. If we deny the existence of specific mechanisms in cell protein aggregation, we will not be able to understand why cell stress initiates such processes as proliferation, differentiation, senescence, apoptosis, necrosis, or mitotic cell death [21]. On the other hand, it is obvious that with all the specificity of interactions leading to protein aggregation, the cellular reaction looks nonspecific because any aggregation, whether specific or nonspecific, ends in the formation of protein complexes. Therefore, it is more correct to focus not on the nonspecificity, but on the *universality *of the complex of structural and functional cellular changes studied by Nasonov's school. That is why I have proposed to name this typical cellular response a universal reaction of the living cell or protoreaction, because there are grounds to consider it the most ancient type of cellular reaction to external actions [8].

Thus, it is the denaturation of proteins that makes these polymers active. Their activity arises from the fact that only denatured proteins begin to interact with each other. This interaction seems to be specific and regular; native aggregation results in new structures that are absent in the resting state and have physiological meaning for the active state. In other words, denaturational changes make proteins *reaction-capable*. While these changes are reversible, the cell is able to disassemble the temporary structures formed and to return to the initial state - the resting state. When damage ensues, when protein aggregation becomes too extensive or irreversible, pathological changes appear in the cell and can lead to its death. The threshold character of the cellular reaction means that the resting state and the active state are different thermodynamic states of the system, which are separated by an energy barrier; this relates not only to the cell as a whole, but also to its individual components [5].

Now the time has come to ask: what makes protein aggregation specific? The answer to this question is provided by the physics of proteins. It has been established that the correct folding of a polypeptide to a globule, like the unique structure of the globule itself, is provided by specific interactions between protein *secondary structures *[7]. Let us consider a structure such as an α-helix. It interacts with other secondary structures via its surface. The surface of the α-helix is "encrusted" with polar (hydrophilic) and non-polar (hydrophobic) groups. Taken individually, these groups are capable only of nonspecific interactions, but the secondary structures confer a specific character on these interactions. This is their biological meaning. Indeed, depending on the amino acid composition, the topography of hydrophobic groups on the surface of an α-helix can vary strongly. If two α-helices have *complementary *topographies of hydrophobic amino acid residues, such secondary structures will "recognize" each other and associate to form a hydrophobic nucleus (the principle of "key"-to-"lock" correspondence works here, too). Owing to the same *topographic *factor, polar groups can form on the secondary structure surface a "landscape" complementary to the nucleic acid surface. To provide specificity of interaction by a unique distribution of protein functional groups on the surface is the main purpose of all protein secondary structures. The principle of structural complementarity has a universal physical basis and is realized not only in intraprotein interactions (in the globular proteins formed and in the process of their folding), but also in interprotein interactions (native aggregation) including protein-nucleic acid interactions.

When an action on a cell or cell structure exceeds the threshold, (i) formation of secondary structures begins in natively unfolded proteins (or unfolded regions of proteins), while (ii) secondary structures of molten globules start to become accessible for interaction with secondary structures of other proteins and with nucleic acids. Such secondary structures induced by the external action are *centers (sites) of native aggregation*. Thus, the first event in the activated cell is the appearance of new secondary structures able to interact selectively with each other to form tertiary, quaternary, etc. structures. Proteins whose secondary structures appear under such circumstances lose their previous inertia and become reaction-capable.

The proposed approach to understanding the mechanisms of cellular reactions poses the question of native and denatured protein states in a new way. In the native state the key cell proteins are inert, non-reaction-capable; they do not interact with each other or with other biopolymers. Loss of the state of inertia is denaturation. On denaturation of the unfolded polypeptide chains the secondary structures appear, whereas on denaturation of molten globules their secondary structures are modified and "float up" to the surface from the hydrophobic nucleus. In both cases the secondary structures are ready to interact. In other words, two extreme protein states can be identified: the completely folded (the globular protein) and the completely unfolded states. Between these inactive (native) states, numerous intermediate, active forms can exist; it is these forms that provide native aggregation. Thus, in proteins, only two states are inactive (they are native states). In all other cases they are active, as manifest in the capacity for native aggregation.

The proposed mechanism of native aggregation explains the increase of volume of the cellular hydrophobic phase during the protoreaction [8] and the structural changes in the universal reaction of the living cell [1]. When secondary structures form, the polar groups of peptide bonds break contact with water and form hydrogen bonds with each other. For this reason alone the hydrophobicity of a polypeptide with secondary structures is higher than in the unfolded polypeptide-precursor. The volume of the hydrophobic phase increases even more when the secondary structures fuse to form hydrophobic domains (nuclei). The second reason why the volume of the cell hydrophobic phase increases further is the appearance of molten globules. In native globular proteins the hydrophobic nucleus is a solid body with a comparatively small surface interacting weakly with hydrophobic substances (therefore, the cell in the resting state is hydrophilic). On melting, the hydrophobic nucleus ceases to be a solid body ([7], Lecture 17); its constituent elements become much more mobile relative to each other, and the nucleus loosens and becomes accessible to water and to substances dissolved in it (surface hydrophobic contacts increase). If the solution contains hydrophobic compounds, it becomes possible for them to penetrate into the molten globule nucleus and become concentrated in this hydrophobic phase.

Proteins in the excited state are capable not only of new intramolecular interactions, but also of interaction with other proteins. Protein physics offers no prohibitions on this point. Native aggregation (formation of specific aggregates) explains the increase of cell turbidity and of macroscopic viscosity of the cytoplasm and nucleus. Thus, the observed changes during the protoreaction are given a simple explanation based on data from protein physics [7].

In this section, significant attention was paid to the cell in the resting state. Let us now consider it in greater detail.

## What is the resting state of the living cell?

To study any process, it is important to identify a starting point. For instance, it would have been impossible to understand the mechanism of muscle contraction without the concept of the resting state of the contractile apparatus. Based on the experience of classical physiology, it is necessary to accept that the concept of the resting state of cell (as well as of its individual parts) is of great importance for understanding the mechanisms of activation. Here we return again to the issue of the structure of the resting cell. The fact that such a cell, unlike an activated one, is almost completely transparent, indicates a negligible amount of protein aggregate. Also, the resting cell is hydrophilic, as under conditions of diffusional equilibrium it does not bind vital dyes [1], which are hydrophobic [8]. These essential peculiarities of the resting cell are to be explained by its structure.

Ling [22] was the first to suggest that the structure of the resting cell is determined by natively unfolded proteins. This concept was finally formulated by 1965 [23], while a summary of the development of this way of thinking was published a decade later [6]. The most important argument in favor of this point of view is the identity of the *equilibrium *distribution of substances between the cell and the medium on the one hand, and between the model systems and the medium on the other. The model systems studied include cellophane dialysis bags filled with concentrated solutions of hydrophilic and electrically neutral *linear *polymers, all of whose chain links are accessible to water. The distribution law, i.e., dependence of equilibrium distribution of substances on their concentration in the medium, is the same for the model systems and for the living cell. Since the distribution of substances was studied under conditions of diffusional equilibrium, this result means that the key physicochemical factor determining the character of the distribution is *identical *in the models and the cell, and is provided by unfolded biopolymers. It seems obvious that of all cell polymers, only proteins - the most massive cell polymers - can possibly fulfill this role [23].

What is this factor? Both cells and models have a common peculiarity: if the solution component studied is not absorbed on a polymer within the system, its equilibrium concentration in the internal medium is always lower than in the external solution. Model systems, owing to their simplicity, allow this phenomenon to be understood: it is because substances are less thoroughly dissolved in the system water than in the water of the external medium. Physics provides the only possible explanation for this difference: water in the cell and in the model systems is more ordered than bulk water; therefore, insertion of a molecule of solute with more rigid bonds into the solvent is not energetically advantageous, so solutes are displaced (excluded) from the system. But why is water ordered in the presence of linear polymers? The obvious explanation is provided by model systems comprising nothing but polymer, water, and dissolved substance: if water is absorbed by the regularly repeated polymer links, the water itself is ordered in the space (multilayer adsorption). Also, in the absorbed water molecules, the electrical properties are different.

In spite of the wide diversity of proteins, they all have absolutely identical polypeptide backbones; differences between proteins are due only to the side chains. The polypeptide backbone of all proteins comprises a regular alternation of positive (NH) and negative (CO) charges in the peptide bonds; the distance between these groups turns out to be comparable with the size of a water molecule and with the length of the hydrogen bonds between them. In other words, the disposition of these dipoles along the polypeptide backbone is complementary to water structure. Another peculiarity of the peptide bond groups is that they form hydrogen bonds either with each other (in the secondary structures) or with water (in the unfolded regions of the polypeptide chain) ([7], Lecture 4). However, the question arises - why does the interaction of water with the functional groups of peptide bonds change its properties so markedly? To answer this question, let us address the properties of electric dipoles.

An important property of dipolar molecules is that their dipole moment is not constant, but depends on their interaction with other dipoles [24]. Example: the dipole moment of water in the gaseous phase is equal to 1.85 D, while in the liquid phase it is 2.9 D. Hence, the interaction of water molecules with each other leads to their mutual polarization - an enhancement of their own dipole moment by 60% [25]. But what if the water molecule interacts with a stronger dipole than itself? The dipole moment of a peptide group is 3.5 D [26]. If water interacts with these, stronger, dipoles, its molecules will be polarized to a greater degree and their hydrogen bonds with other molecules will become stronger. The enhancement of hydrogen bonds makes the first adsorptional layer stable and able to attract and to bind more and more new free water molecules, forming more and more new adsorbed layers. Thereby, stronger dipoles on the adsorbing surface are the key prerequisites for the multilayer adsorption of polar molecules.

Owing to the enhancement of hydrogen bonds in the multilevel adsorbed water layer, penetration of other molecules into it (including water itself) becomes energetically non-advantageous, because it requires breakdown of the intermolecular hydrogen bonds in the layer, which are stronger than in the voluminous (bulk) phase. This explains why bound water is a poor solvent compared with the phase in which water molecules interact only with each other. For this thermodynamic reason, the concentration of *any *substance in the absorbed phase always will be lower than in the liquid phase.

However, all begins to change if the unfolded polypeptide absorbing water begins to fold with formation of secondary structures. In this process, peptide groups cease to form hydrogen bonds with water and form them between each other. The previously bound water is desorbed and acquires the properties of voluminous (bulk) solvent [6,23,27]. There is convincing experimental evidence to substantiate this point of view about the interaction of polypeptides and other hydrophilic polymers with water [28,29].

But what is the role of globular proteins? It is these compounds that are the second important component of the cell in the resting state. They are the best-studied type of proteins, performing structural and enzymatic functions. Their solid core is inaccessible to water, while polypeptide chains containing no secondary structures are not sufficiently expanded to affect the state of the intracellular water fundamentally [5].

Thus, in the resting state, the physical properties of the cell protein matrix are determined by partially or completely unfolded proteins and by globular proteins (of course, the latter include complex proteins with several globular domains). In the context of the present paper, such proteins can meaningfully be called native. The structural and functional peculiarities of the cell in the resting state are determined by unfolded proteins [5].

The question remains as to why the resting state of the cell is relatively stable and can exist for an indefinite period. Ling believes this is accounted for by the stabilizing effect on unfolded proteins of various ligands bound to native unfolded proteins: ions, low-molecular organic compounds, hormones, etc. According to Ling, the most important ligand of proteins in the resting state is ATP [30]. If some action leads to splitting of ATP or to dissociation of other rest-making ligands, this leads to folding of the natively unfolded protein; secondary structures appear and make the polypeptide reaction-capable. Native aggregation begins, in the course of which signaling structures are formed. Natively unfolded proteins seem to be the most sensitive elements of the resting cell, as their folded state is economically advantageous, because when the water is desorbed the entropy of the system increases (water is the most abundant cell component). Also, the rest-making ligands are not firmly bound to natively unfolded proteins, as the bonds are non-covalent, while ATP can be split enzymatically. As a result, individual cell components or the entire cell appear as a system in which the structural content of life activity is the reversible transition from the resting state into the activated (excitatory) state provided by the reversible transition of proteins from the resting (native) into the activated (non-native) state.

## Principles of native aggregation

From the point of view of the proposed approach, reactions of the cell to external actions, various forms of cellular activity (metabolism, division, muscle contraction, secretion, intracellular signaling, etc.) as well as pathological states are considered on the basis of the following statements and principles.

Native aggregation is a specific interaction of proteins with each other, realized by interaction between the secondary structures of the aggregating proteins. If the reaction-capable secondary structures are absent or inaccessible for interaction, native aggregation is impossible.

The cell is considered as a system that can have only two states: the resting state and the active (excitatory) state. The same principle is true for any cell organelle, structure or protein molecule. For clarity, a parallel can be presented: the excitable membrane in a state of rest or excitation.

Functionally important cell proteins in the resting state are present in one of two states: either unfolded (completely or partly - natively unfolded proteins) or folded to the protein globule state or any other form in which secondary structures are inaccessible for interaction with other proteins. These states are considered the resting states of protein molecules or as their native states. Proteins in the native state are stabilized by rest-making ligands and/or chemical modifications, for instance, by phosphorylation/dephosphorylation. According to Ling [30], the resting state of an unfolded protein is maintained by its bound ATP, ions (Na^+^, K^+^, Ca^2+^), molecules of bound water, hormones (for instance, insulin), and any other significant interactions. For instance, analysis of amino acid sequences in the regions surrounding known phosphorylation sites reveals a strong propensity towards adoption of a natively unfolded conformation [31]. Disruption of bonds with ligands (for instance, breakdown of ATP) leads to activation of the protein, its transition from the resting to the active state; the same result is produced by a decrease in the cell ATP content below the critical level. Ling's concept of the capability of small molecules for specific binding with natively unfolded proteins is confirmed, for instance, in the work by Mukhopadhyay et al. [32].

**Figure 2:**
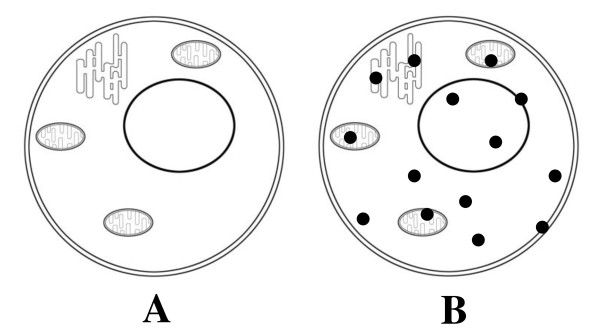
**Two main cell states**. A - cell in the resting state, optically transparent. B - activated cell, in the cytoplasm and organelles of which native aggregation centers (closed cycles) appear - the reaction-capable secondary structures of activated proteins, owing to which native aggregation of cell proteins begins.

On activation of the cell by external actions, intracellular factors, and signals of different nature (including chemical modification), a new protein fraction appears -- activated proteins with newly formed secondary structures that were absent in the resting state (Fig. 2). These new structures appear on the folding of natively unfolded proteins and on melting of protein globules. They include α-helices, β-sheets, and other secondary structure variants. The secondary structures of activated proteins are new "valences" necessary for new interactions - intramolecular (folding) and intermolecular (native aggregation). In the case of large proteins, the secondary structures can form hydrophobic sites on the protein surface, which interact specifically with similar (complementary) structures on the surfaces of other proteins.

The natively unfolded proteins can be called excitable proteins. Their transition to the excitatory state triggers native aggregation.

If on the unfolding of a globule (or a globular domain) no molten globule intermediate is formed, while the protein cooperatively assumes the completely unfolded configuration at once, this means it is inactivated, as a protein without reaction-capable secondary structures is incapable of aggregation. The molten globule may be inactivated in two ways: by transition back to the well-folded conformation (when secondary structures are hidden from interaction) or by unfolding of the molten globules until a completely unfolded conformation is reached, devoid of the secondary structures that are key to native aggregation.

The secondary structures in activated proteins play the role of centers of native aggregation. It is these structures that provide for specific interactions of activated proteins with each other (native aggregation) to form new structures that have signaling and functional significance for the active cell. Native aggregation is determined by the same forces and interactions that are involved in the well-studied folding of unfolded polypeptides to globules. This rule is followed: if there are secondary structures capable of specific interaction, there is native aggregation; if there are no such structures or they are inaccessible, there is no native aggregation.

If an action on a protein increases the number of amino acid residues included in its secondary structures, that protein is activated and the signal pathways in which it participates are open. If the protein is unfolded and the portion of amino acid residues in secondary structures decreases, it undergoes transition to the inactive state, is relaxed, while the signal pathway(s) in which it participate(s) is/are blocked. On the transition of proteins participating in native aggregation to the native state, native aggregates are destroyed and individual structures and the cell as a whole transit to the resting state.

The temporary structures appearing as a result of native aggregation perform diverse functions. They may be centers of specific adsorption (binding) of various ions and molecules including signal factors and proteins, i.e., can perform the functions of receptors. They may have the enzymatic activity necessary for performing specific functions and may serve as centers of formation of even more complex supramolecular structures.

Only the secondary protein structures are able to provide for specificity (selectivity) in the interaction of proteins with others, as they provide the specificity of interactions necessary for correct folding of the polypeptide chain to a globule (the folding of polypeptide to native globule can be considered as intramolecular native aggregation). Each secondary structure has a unique topology of polar and hydrophobic groups on its surface. Secondary structures form stable complexes with each other or with sites on nucleic acids only if their surfaces are complementary to each other, as the key is complementary to the lock.

Native aggregation is determined genetically to the same extent as protein structure because it is determined by the same factors that determine all levels of organization of the individual protein molecule beginning with the primary sequence. Secondary structures of activated (excited) proteins will interact with other excited proteins not chaotically, but in accordance with the genetic program. As a result of native aggregation, those structures and corresponding functions will appear that are necessary to the cell here and now: action potential, channels on the cell surface, in the cytoplasm and nucleus, cytoskeleton, movement of cytoplasmic sites, cell division, apoptosis. Errors in native aggregation that appear during a prolonged state of cell excitation (for instance, chronic inflammation) and on damage lead to various forms of cellular pathology: conformational diseases, necrosis, carcinogenesis.

All the differences between the excited cell and the cell in the resting state are the direct or indirect results of native protein aggregation.

## Native aggregation in action

Since practically any change in the cell can be considered a result of native aggregation, I will focus on only a few examples. The aim of this section is to show how the principles of native aggregation work in the analysis of particular phenomena.

I will begin with the natively unfolded proteins, the physical basis of the resting state. According to Dunker et al. [33], the first data about natively unfolded regions in proteins appeared in 1978, i.e., 26 years after Ling [22] had first suggested their existence. Until the discovery of natively unfolded proteins, the dominant notion was that the whole diversity of cell functions is due only to proteins with 3D structure. Natively unfolded proteins were not compatible with this notion and it was not clear whether they performed any function at all. Subsequently it was found that more than 35-51% of eukaryotic proteins had unfolded regions longer than 50 consecutive amino acid residues, which is significantly higher than in prokaryotes [34,35].

When it became clear that natively unfolded proteins played an important role, Dunker et al. [33] proposed to widen the notion of functional protein types in the cell: to proteins with 3D structure, they added molten globules and proteins with unfolded conformations. Uversky [36] proposed to supplement this list with a fourth, relatively stable protein conformation - the premolten globule, which might be called the boiling globule, as in the coordinates of the unfolding reaction it follows the globule and molten globule and precedes the completely unfolded conformation. The rationale of this proposal is that all four protein states are thermodynamically stable, although to different degrees.

In the opinion of Dunker et al., transitions between different phasic states continually take place in the cell. This is so, indeed; however, the statement needs clarification. Let us recollect that the first ideas about the molten globule and unfolded protein conformation were obtained by studying protein denaturation *in vitro *and then they were extrapolated to the cell. Nowadays we know that globule melting is a phase transition that fits the "all-or-nothing" law and has a threshold, for instance, a temperature threshold [7]. This means that several similar molecules under identical conditions will be in the same phase state: either globule, or molten globule, or the unfolded conformation. Within such a population, uninterrupted and asynchronous protein transitions from one phase state to another cannot take place. However, molecules of the same protein located in different microenvironments can be in different phase states, but the state may also be identical for all proteins of the same (given) population. As a result, we find that this (some) protein can indeed be in *different *phase states in this cell, but only if its molecules are located in *different *parts of the cell with different microenvironmental conditions.

Another specification is also to be made. According to the hypothesis of native aggregation, there are only two basic protein states in the resting cell: globules (here, proteins composed of two and more globular domains may be included) and the natively unfolded state. Other transitional states appear in the cell temporarily. They appear on reaching the threshold, when some factor in the medium begins to produce a moderately (gentle) denaturing action. Then a globular protein is melted, after which it unfolds (if the strength of the action keeps rising), while natively unfolded proteins begin to fold. The differences between the main states are fundamental: the globular conformation is stabilized mainly by hydrophobic interactions, the natively unfolded one by ATP and other ligands. As soon as the medium conditions return to normal, the excited proteins are relaxed and the system returns to its main state - the resting state.

Since native aggregation results in the appearance of signaling and regulatory structures, it is obvious that as biological organization becomes more complicated during evolution, more and more novel mechanisms of regulation of the active cell are needed. This need is realized with the aid of new natively unfolded proteins and, accordingly, of new transitory conformations appearing as they fold.

In the literature, the mechanism of interaction of natively unfolded proteins with protein targets has been widely discussed. Most commonly, four stages of such interaction are identified: (i) random collision of natively unfolded protein with target; (ii) weak, nonspecific interaction of natively unfolded protein with target; (iii) formation of secondary structures in natively unfolded protein; (iv) owing to these nascent secondary structures, a firm complex of the natively unfolded protein with the protein-target is formed [37,38].

In terms of the hypothesis of native aggregation, this scheme looks unconvincing. Indeed, it is hard to imagine a mechanism (for instance, the mechanism of muscle contraction) or a process in the living cell working on the basis of random collisions. First, if natively unfolded proteins and their targets collide randomly, it means that they are diffusing freely in the cytoplasm or nucleus, i.e., we are dealing with a Brownian mechanism of regulation. Second, if the first stage of interaction of the natively unfolded protein with the target is accepted as nonspecific, this will mean that the number of interactions of the diffusing natively unfolded protein will greatly exceed the number of interactions necessary for the act of regulation. Under such conditions, the correct regulatory response looks more random than regular.

From the point of view of native aggregation, these events appear differently. The available experimental data indicate that natively unfolded proteins are organized in clusters and oriented in space mainly in parallel to each other ([5], Ch. 11), while the protein concentration in the cytoplasm reaches 200-400 mg/mL [39]. Thus, under conditions of crowding, when the space between protein molecules is not large and is filled with bound water ([5], Ch. 11), it is difficult to imagine diffusion of free proteins. According to Ling, the protein matrix of the resting cell is not chaotic, but structured. In terms of the hypothesis of native aggregation this means that the program of protein-protein interactions is responsible for the spatial distribution of the key matrix elements (for instance in the contractile apparatus). Natively unfolded protein does not diffuse in anticipation of a random hit to the target. The target is relatively immobile and is located nearby. In the resting state they do not interact with each other, as they are in the inactive (native) state, i.e., do not have reaction-capable secondary structures.

If secondary structures are formed in the natively unfolded proteins during any collision with other proteins, this will also become a random event and the interactions of secondary structures with each other will not be amenable to any logic. For this reason, random, nonspecific interactions are to be eliminated from the mechanism of functioning of natively unfolded proteins. To prevent random interactions from causing excitation of the natively unfolded state, such proteins must be sufficiently stable. According to the proposed approach, the natively unfolded proteins are stabilized by various ligands depending on their property, location, and function [6].

The fourth stage of interaction with the target is also problematic because the *activated *native protein will interact, in my opinion, only with *activated *protein-target (with its active secondary structures). Native globular proteins (or globular domains in large proteins) in the native state do not have secondary structures accessible for external interactions. This is prevented by the rigid nuclear structure of such proteins ([7], Lecture 13).

Thus, we see that the hypothesis of native aggregation differs from the model accepted in the literature in that it involves nothing random and nonspecific. Moreover, it contains elements of control and management: genetic control of the primary sequence (hence, also the properties of secondary structures), ligands, highly specific interactions of secondary structures with each other, and spatial control of the course of native aggregation.

As for spatial control, it is also provided first of all by interactions of "residual" secondary structures of neighboring natively unfolded proteins (from the point of view of the proposed approach). This is quite a substantive suggestion, if we take into account that the complete absence of secondary structures is possible under the most severe conditions ([7], Lecture 17). If we also take into account the selective binding role of "residual" secondary structures, the spatial structure of the protein matrix in the *resting state* is also under genetic control, as properties of the "residual" secondary structures are encoded by the primary sequence of amino acid residues.

Now let us consider the properties of a molten globule in greater detail. Packing of polypeptide chain of normal globule is dense that the side chains are tightly apposed to each other and their rotation around valence bonds (turn isomerization) is impossible. When the nucleus melts, the globules increase in volume by approximately 50% [36]; free volume appears and, concomitantly, turn isomerization also becomes possible. As a result of nuclear loosening, water and hydrophobic substances (for instance, the dye ANS) begin to penetrate into the nucleus. If the intensity of the denaturing factor rises, the molten globule is converted into a premolten globule, in which the amount of secondary structure is approximately half that in the molten globule ([7], Lecture 18).

These properties of the molten globule (to say nothing about the premolten one) indicate that its nucleus loses rigidity and more closely resembles a fluid. An elevation of conformational temperature inevitably leads to increased mobility of parts of the molecule and to a decrease of the portion of the polypeptide chain included in secondary structures. Modification of secondary structures inevitably leads to a change of their specificity due to a change of their topological characteristics. In other words, a change in size of secondary structure (for instance, length of α-helix) means a change in the biological meaning of the polypeptide "sentence". The logic of this statement has been confirmed experimentally in studies indicating that the nucleus of a molten globule is structurally labile [40]. Thus, the molten globule is converted into a reaction-capable protein that can participate in native aggregation.

Next, let us consider data indicating the involvement of the protein secondary structures in mechanisms of signal transmission. Kim et al. [41] studied the dynamics of the cytoplasmic domains of the *E. coli *chemotaxis receptor on interaction with repellent and attractant. These authors concluded that an attractant decreases the number of secondary structures in the domain, which blocks signal transmission into the cytoplasm. A repellent produces the opposite effect: it increases the amount of secondary structures in the domain, and this makes the signal function of the receptor possible. In terms of the hypothesis of native aggregation, repellent converts the domain into the excited state, when its "valence" for interactions necessary for signal transmission appears. The authors also believe that methylation/demethylation of receptors is so important for their clustering and the dissociation of the formed clusters because it causes significant changes in the amount of secondary structures in domains.

Williams et al. [42] note that the orderliness of a polypeptide chain is closely connected with protein function. Thus, for instance, binding of ligands to streptavidin, purine nucleoside phosphorylase, hypoxanthine-guanine phosphoribosyl transferase, hemoglobin, and myoglobin leads to some disorderliness in the protein molecules. The authors performed thermodynamic analyses of the actions of agonists and antagonists on the corresponding receptors and came to the conclusion that mechanism of action of these ligands was connected to opposite effects on the orderliness of the receptor structure; denser polypeptide chain packing inside the protein leads to enhancement of the degree of receptor oligomerization, while less dense packing decreases the degree of oligomerization. Interestingly, agonists produce opposite structural changes in different receptors. Thus, while an agonist of receptor 1 increases polypeptide chain packing in receptor 1, an agonist for receptor 2 decreases the packing in receptor 2. The same principle applies to antagonists. The physiological sense of these changes will be understood only when it becomes clear which part of which signaling pathway these changes constitute. Receptors are one more system for which the existence of two states - resting and activated - seems obvious. In this sense, the cell may be considered a megareceptor: conversion from one state into another produces a complex signal to neighboring cells.

According to current concepts, chaperones play an important role in cell life. An example of interest is the small heat-shock proteins, a variable class of chaperones widely distributed in cells of various types. Some representatives of this family are inactive in cells in the resting state and are activated, for instance, on heating [43]. According to the logic of native aggregation, the triggering action of heat not only leads to the appearance of non-native protein forms, but also activates the heat-shock proteins themselves. For this, they must necessarily have either natively unfolded polypeptide chain regions or the ability to be converted into the molten globule state. This will lead to formation of a native aggregation center and then to aggregation itself. Native aggregation of an *activated *chaperone with an *activated *target begins.

The presence of natively unfolded regions in chaperone molecules has been accepted in the literature as necessary for their work [44-46]. From the point of view of native aggregation, these unfolded sites are needed for the formation of the secondary structures necessary for native aggregation with a target. But the target itself is an excited protein that can become either a natively unfolded protein (or have natively unfolded protein regions) or a globule that becomes a molten globule. This suggestion is confirmed by the studies of Hegyi and Tompa [47], who showed that natively unfolded proteins have no tendency to interact with chaperones. This result is understood. Natively unfolded proteins are proteins in the resting state. To interact with other proteins, including chaperones, they must be activated - to be converted into the excited, denatured state. On the other hand, chaperones have long been known to be able to interact with molten globules [48].

From the point of view of the proposed approach, the results of native aggregation are new structures necessary for the excited cell. The formation of such structures is a cooperative process that needs the participation of two or more proteins. Without such cooperation, the new structure cannot be created. With such an understanding of native aggregation, it becomes obvious that each of the two or more proteins, when interacting with each other, helps the correct folding of the protein-partner. In other words, all proteins participating in native aggregation are chaperones for each other, but some of them might be more profoundly specialized in this direction.

It is well known that for the release of protein-targets, some chaperones need ATP [43]. This fact is well explained in terms of Ling's concept: binding of ATP leads to disassembly of the secondary structures formed in the natively unfolded regions of the chaperone molecules bound to the protein-target. As a result, the complex of chaperone with target is split. For other chaperones, the role of ATP can be played by different ligands.

Native aggregation, like any other process in the cell, can be an object of regulation. Its course can be affected by various factors that produce new signaling structures. As an example, programmed cell death can be considered. The mechanism of genetic regulation of apoptosis can be a source of the signal that leads, as a result of native aggregation, to the appearance of a structure that will trigger the whole cascade of reactions necessary for cell degradation. From the point of view of native aggregation, such a structure can appear in any part of cell - in the nucleus, cytoplasm, organelles, or plasma membrane. The "structure of death" produced by native aggregation can also appear if the cell (or any of its parts) is damaged. By the same mechanism, other cell pathologies, for instance cancer, can appear.

With reference to the peculiarities of cancer cells, I would like to note one feature that is directly related to the subject of the present paper. The content of bound water in cancer cells is known to be lower than in precursor cells [49]. It is on the basis of this difference that the technology of magnetic resonance imaging allows malignant tumors to be recognized non-invasively. From Ling's point of view, this means there are fewer natively unfolded proteins in the cancer cell than in the normal cell. At the same time, it has been shown *in silico *that the natively unfolded regions are more extensive in cancer cell proteins: in cancer-associated proteins, the number of such areas is 70% greater, while in signaling proteins it is 5 times greater [50]. It is obvious that natively unfolded proteins represent a diverse population and are directly involved in cell transformations of pathological character.

## Dynamics of the hydrophobic phase of the living cell

As I already mentioned, the cell in the resting state is a hydrophilic system. This is confirmed by data on the distribution of hydrophobic substances (vital dyes) between cell and medium *under conditions of steady-state distribution*: the cell in the resting state does not adsorb such substances [1].

Here I would like to draw the reader's attention to a very important circumstance: under conditions of diffusional equilibrium, the plasma membrane stops working as a barrier to a diffusing substance. There are no absolutely impermeable membranes, especially for hydrophobic substances. The dye undoubtedly penetrates into the resting cell, but is not accumulated in it. Why? There are two reasons: in the cell, hydrophobic binding centers for dyes are absent; and intracellular water is a poor solvent for them. For these reasons, the dye molecules penetrating into the cell are eventually pushed out into the medium. Thereby, under conditions of *steady-state distribution*, the character of the distribution of the substance between cell and medium is determined by only two factors: sorption on intracellular structures, and the low solving capability of intracellular water ([51], Ch. 5).

Everything changes when the cell is converted to the excited state: binding of vital dyes under conditions of steady-state distribution rises by tens or hundreds percent of times [1]. Only one explanation for this is possible: the volume of the hydrophobic phase in the cell increases explosively [8].

The hydrophobic phase is habitually associated with the membrane lipid phase; but the volume of the lipid phase is negligible compared with cell size and, what is most important, it cannot rise tens of times in fractions of second. However, as we already know, proteins in the excited cell undergo denaturational changes [1]. Hence, the cause of the increase of cell hydrophobicity should be looked for in proteins rather than in lipids [8].

The hypothesis of native aggregation provides a simple explanation for the hydrophobic burst in the cell: the cause of the increase of the hydrophobic phase is the appearance of excited proteins. Indeed, according to the proposed approach, when the threshold of perturbating action on natively unfolded proteins is exceeded, secondary structures begin to form. These structures, in the course of native aggregation, are then included in the hydrophobic areas of new structures - structures of excitation. As stated earlier, the hydrophobic areas are formed not only by molten globules, but also by the secondary structures appearing on the folding of unfolded protein regions.

The high rate of formation of secondary structures, within the microsecond time range ([7], Lecture 9), also determines the high rate of native aggregation overall, which explains the hydrophobic burst in the excited cell. On the reverse transition to the resting state, the cell again becomes hydrophilic. According to the hypothesis of native aggregation, significant changes in the hydrophobic phase can take place in any cellular structure, including membranes and organelles.

The proposed existence of temporary hydrophobic protein phases explains interesting phenomena known from pharmacology, when the efficiency of a therapeutic agent depends on the degree of functional activity of the target cell. The best known example of this seems to be verapamil. This hydrophobic compound [52] scarcely affects the normal heart rhythm, but very efficiently inhibits tachycardia. The same regularity is also observed in the action of verapamil on skeletal muscle. This dependence can be explained if, on excitation, verapamil-binding hydrophobic receptors appear in the muscle fiber. The effect of verapamil is due to its blocking action on slow calcium channels; but from the point of view of the principles of native aggregation, the cell can also contain other dynamic hydrophobic targets for pharmacological agents of various types. In other words, using the native aggregation principles, it is possible to predict the existence of drugs acting only on the active cell; their targets can be located not only in the membrane (as in the case of verapamil), but also in other parts of the cell. Such medications will produce no marked effect on cells in the resting state (the healthy state).

The role of the dynamic hydrophobic protein phase in the life of the cell has not been studied at all. It is unknown in the equations of cell physiology. At present, one can discuss the significance of this X-factor only in terms of very general regularities based on simple physical principles. For instance, it is obvious that the appearance of the hydrophobic phase in a cell will cause the redistribution of *all *hydrophobic compounds including ATP [8]. The redistribution of hydrophobic substances between the cell and the medium will also begin.

However, the redistribution of substances is triggered not only by the appearance of the temporary hydrophobic phase, but also by the desorption of water from protein surfaces. As secondary structures start to form, the adsorbed water will become free and the "bad" solvent will become "good". This will lead to a rapid invasion of small solute molecules into the areas that were previously occupied by adsorbed water. If we take into account the rapid rate of formation of secondary structures ([7], Lecture 9), it becomes obvious that during the fast destruction of the ordered water structure, sharp concentration gradients of such substances will appear. In the case of ions, everywhere in the cell, in microvolumes, significant diffusional potentials will appear that may prove to be one cause of the appearance of molten globules. Significant concentration gradients of dissolved substances can also appear when the ordered water layers are restored, as the rate of their restoration will also be determined by the high rate of disassembly of secondary structures in activated proteins.

It is obvious that during the course of native aggregation the density and rigidity of the protein matrix will increase owing to a rise in the number of interprotein contacts. This provides even more difficulties for models of cell function regulation that base their mechanisms on the free diffusion of substances in the cell, since with an increase of protein matrix density the significance of diffusional processes will decrease.

If we return to the cell protoreaction, it can be concluded with certainty that the hypothesis of native aggregation has managed to explain the rise of viscosity and turbidity of the cytoplasm (Fig. 1) as well as the increase of volume of the cell hydrophobic phase. From the proposed mechanism it is clear that the changes discussed will occur synchronously, as the key link among all these changes is the structural readjustment of the same key proteins.

## Conclusion

The cornerstone of the hypothesis of native aggregation is the generation in proteins of temporary secondary structures that can interact selectively with secondary structures in the same or other proteins. The nonspecific reaction of cells, which was studied by Nasonov's school, turns out in reality to comprise myriads of specific protein-protein interactions. Since native aggregation is directed by active secondary protein structures, it proves to be completely under genetic control, so the dogma of Anfinsen [53] formulated for the folded polypeptide chain can be extended by incorporating native aggregation into its sphere of application.

## Competing interests

The author declares that they have no competing interests.
